# Who Needs Cause-of-Death Data?

**DOI:** 10.1371/journal.pmed.0040333

**Published:** 2007-11-20

**Authors:** Peter Byass

## Abstract

The author discusses two studies that report important methodological advances in determining cause of death, which is crucial for health planning and prioritization.

More than half of the world's deaths pass by undocumented as to cause [1]. Whilst the appropriate focus of health services may well be the care of the living, consistent and reliable cause-of-death data also constitute a crucial and major resource for health planning and prioritisation, and their lack in many settings is a major concern. Two new papers from Christopher Murray and colleagues in this issue of *PLoS Medicine* [2,3] report important methodological advances which should go some way towards filling these data gaps.

## The Inequalities of Dying

To paraphrase George Orwell, all deaths are equal, but some are more equal than others. In particular, the chance of a death being registered and documented as to cause depends strongly on the socioeconomic status of the community and nation in which it occurs, and this is a major obstacle in coming to a meaningful global overview of mortality patterns.

Whilst richer settings have traditionally aggregated physician death certificates and autopsy data as the basis for public health reporting, in poorer circumstances alternative approaches have to be used. Over the last 25 years, these strategies have often involved so-called “verbal autopsy” (VA)—interviewing relatives and witnesses of deaths and interpreting the interview material to arrive at cause(s) of death [4,5].

Much VA interpretation has been undertaken by physicians (physician-coded verbal autopsy, PCVA), but this approach makes large demands on limited resources and can be inconsistent over time and place. Much work on VA methodology has concentrated on emulating individual physician death certification, often glossing over the considerable variability and imprecision with which death certificates, the supposed “gold standard,” are sometimes completed [6].

**Related Research Articles**

This Perspective discusses the following new studies published in *PLoS Medicine*:

- Murray CJL, Lopez AD, Barofsky JT, Bryson-Cahn C, Lozano R (2007) Estimating population cause-specific mortality fractions from in-hospital mortality: Validation of a new method. PLoS Med 4(11): e326. doi:10.1371/journal.pmed.0040326
  Working in Mexico and using vital registration data, Chris Murray and colleagues achieved encouraging results with a new method to estimate population cause-specific mortality fractions.
- Murray CJL, Lopez AD, Feehan DM, Peter ST, Yang G (2007) Validation of the symptom pattern method for analyzing verbal autopsy data. PLoS Med 4(11): e 327. doi:10.1371/journal.pmed.0040327
  Chris Murray and colleagues propose and, using data from China, validate a new strategy for analyzing verbal autopsy data that combines the advantages of previous methods.

Newer approaches using computer models for interpreting VA data are now tending to supersede PCVA, both for populations in general [7,8] and for specific subgroups [9,10], putting more emphasis on cause-specific mortality fractions (CSMFs) than on individual causes.

## Who Really Needs What?

Methodological advances in cause-of-death determination have not always been explicit about which gaps in the global data they seek to fill, and this has sometimes led to a confused overall picture. There are different levels at which data on mortality patterns are needed (i.e., from the local to the global) and various ways of meeting these needs, as shown in Table 1.

**Table 1:**
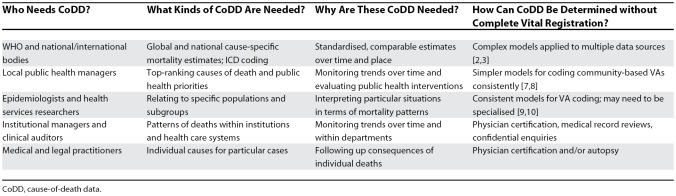
Cause-of-Death Data: Who, What, Why, and How CoDD, cause-of-death data.

Murray and colleagues' new approach for estimating population CSMFs [3] within countries that have existing data on hospital deaths and partial vital registration is a big step forward from simply reporting facility-based data. Although it still depends on the availability of requisite data, it represents an important way forward for understanding mortality in transitional countries, without needing primary data capture.

Their other paper [2] is a further development in the trend away from PCVA towards more cost-effective and consistent approaches to VA interpretation, with examples from China. Refinement of VA approaches remains a very important area of methodological development for settings where VA is the only realistic source of cause-specific mortality data, particularly in sub-Saharan Africa. However, applying this more sophisticated approach to VA interpretation globally would still require a large international database of symptom-level sensitivities.

These new papers from Murray et al. can thus be contextualised as potentially filling important gaps at the global level, but other gaps will remain at various levels, requiring their own particular solutions. WHO has recently finalised a framework for an internationally standardised approach to VA integrated with the International Statistical Classification of Diseases and Related Health Problems, Tenth Revision (ICD-10) [11]. This new integrated approach is another major contribution at the global level, also making the case for VA-based approaches rather than post-hoc modelling of available mortality data into overall estimates.

It is important here to distinguish clearly between using computer models to interpret case-by-case VA material (on which the widespread future utility of VA depends) and the direct modelling of mortality statistics (which is a second-best approach in the absence of detailed data). WHO's approach should also facilitate comparability between VA and aggregated death certificate data sources; however, ICD coding was not conceived primarily as a public health tool, and it may not be the best means for identifying local public health priorities from cause-of-death data. Tools enabling local health managers to readily monitor mortality patterns and identify priorities in their own local areas remain scarce.

## Completing the Picture

Realistically, there will not be universal vital registration and individually based cause-of-death data on a worldwide basis anytime soon, no matter how useful such information might be in public health terms. Therefore a mixed-methods approach will continue to be used, combining data sources that are most appropriate to their particular settings, and meeting needs at different levels.

Basing CSMF population estimates on hospital death data as proposed by Murray et al. [3] is a novel example of using existing data to fill information gaps. However, as with other approaches, Murray and colleagues' approach is context-dependent (requiring a reasonable proportion of deaths to occur in hospitals). Consistency and comparability are crucial aspects of combining data from a range of sources into a bigger picture, as well as an essential basis for monitoring trends over time. It is likely that further advances in computer models for interpreting cause of death from VAs will contribute by attaining greater accuracy, while inherently avoiding the vagaries of inter-observer subjectivity.

Further thinking on the “cause-of-death” concept in public health terms, in addition to the traditional medical model, may also lead to helpful advances. For example, if a woman dies as a consequence of prolonged, obstructed labour during a period in which no medical personnel nor ambulance driver was available at her local health centre, it could be argued that the public health cause was “health systems failure.”

The traditional structure of immediate, underlying, and secondary medical causes of death may also be less relevant to public health. More relevant is the concept that a particular death could have been due to two or three alternative causes that are not interdistinguishable on the basis of the available evidence but can each contribute fractionally to population CSMFs.

## Conclusion

Today's world is a long way from having the comprehensive picture of mortality patterns needed for effective health planning. Murray and colleagues' new methods make important contributions to filling some gaps at the global level, but further methodological development and wider support for implementing cause-of-death surveillance are still needed at all levels in the world's poorest nations.

## Abbreviations

CSMF: cause-specific mortality fraction
PCVA: physician-coded verbal autopsy
VA: verbal autopsy
